# Supplementary material for: Complexome profiling of the Chlamydomonas psb28 mutant reveals TEF5 as an early PSII assembly factor
Source: Plant Cell. 2025 Mar 17;37(6):koaf055. doi: 10.1093/plcell/koaf055 (PMC12164590; doi:10.1093/plcell/koaf055)
Supplement: koaf055_Supplementary_Data [file koaf055_supplementary_data.zip › psb28_interactive.zip!/plots/intensity/qualityPlots/peptides/_Cre09.g401886.t1.1.html]

### Cre09.g401886.t1.1

Trivial name:   
SeqCoverage [median]: 41.40  
Mass: 39.45 kDa  
  
 

| Sequence | MissedCleavage | Mass [Da] | wtIntensity | muIntensity | Score |
| --- | --- | --- | --- | --- | --- |
| AAAEAPAAAAAAASK | 0 | 1240.64 | 1109.78 | 3552.63 | 122.70 |
| AAAEAPAAAAAAASKDEAK | 1 | 1683.84 | 22783.80 | 28712.51 | 100.77 |
| DEEAFFKDFAVAFSK | 1 | 1749.83 | 550.68 | 0.00 | 45.33 |
| IVPDGRLPDAK | 1 | 1179.66 | 16887.20 | 16331.87 | 59.43 |
| LAWHASGTYAK | 0 | 1203.60 | 562.99 | 0.00 | 51.93 |
| LLELGVPFPAK | 0 | 1182.70 | 16596.35 | 0.00 | 124.23 |
| MGFDDKDIVALSGAHTLGR | 1 | 2001.99 | 0.00 | 8954.84 | 73.11 |
| PSTPGDYAAVR | 0 | 1132.55 | 884.32 | 2651.01 | 96.33 |
| QSIAALLDDGDYDDGSYGPVLVR | 0 | 2438.16 | 1543.29 | 0.00 | 42.32 |
| SQSLMMLPSDLALLSDR | 0 | 1875.94 | 769.23 | 0.00 | 142.45 |
| VPESGPLAGPPALVTSKPSTPGDYAAVR | 1 | 2733.43 | 10462.18 | 12534.39 | 114.01 |
|  |  |  | 72149.8 | 72737.3 |  |
